# Factors Influencing Hospital Non-clinical Personnel’s Willingness to Perform Bystander Cardiopulmonary Resuscitation in the Immediate Post-COVID-19 Period

**DOI:** 10.7759/cureus.90524

**Published:** 2025-08-19

**Authors:** Jong Sun Ok, Soo Young An, Gukhee Lee

**Affiliations:** 1 Department of Nursing, Konkuk University, Chungju-si, KOR; 2 Emergency Department, Konkuk University Medical Center, Seoul, KOR; 3 Medical Innovation Department, Konkuk University Medical Center, Seoul, KOR

**Keywords:** cardiopulmonary resuscitation, hospital non-clinical personnel, out-of-hospital cardiac arrest, post-covid-19, willingness

## Abstract

Background

Out-of-hospital cardiac arrest (OHCA) is a major emergency that requires immediate intervention, and the willingness of bystanders to perform cardiopulmonary resuscitation (CPR) plays a crucial role in improving patient survival. This study aimed to investigate the factors associated with the willingness to perform bystander cardiopulmonary resuscitation (BCPR) in the immediate post-COVID-19 pandemic context.

Method

A descriptive cross-sectional survey was conducted among 215 hospital non-clinical personnel employed at Konkuk University Medical Center in 2023. Data were analyzed using multiple linear regression to identify key predictors of willingness to perform BCPR.

Results

The findings indicated that willingness to perform CPR was lowest when the victim was a trauma patient and highest when the victim was a family member. Contrary to expectations, fear of infectious disease transmission did not significantly deter CPR willingness. Instead, concern about potentially harming the victim emerged as a major psychological barrier. Factors positively associated with increased willingness to administer BCPR included prior CPR training, higher self-reported confidence in CPR performance, and female gender.

Conclusion

In light of the ongoing and possibly recurring nature of infectious disease outbreaks, it is imperative to develop multifaceted public health strategies aimed at enhancing public readiness to perform CPR. Improving CPR education, addressing psychological barriers, and reinforcing the importance of bystander intervention are essential to improving OHCA survival rates and optimizing health system preparedness and community resilience.

## Introduction

Out-of-hospital cardiac arrest (OHCA) remains a significant public health concern worldwide [1]. Prompt initiation of bystander cardiopulmonary resuscitation (BCPR) is recognized as a critical determinant of survival, significantly increasing the likelihood of favorable outcomes for OHCA patients [2]. The willingness of laypersons to initiate BCPR is a vital component in the chain of survival, as immediate response and participation can more than double survival rates [1].

However, the COVID-19 pandemic had a substantial negative impact on the implementation of BCPR. Globally, the BCPR rate declined from a pre-pandemic high of 55.2% to 27.6% during the pandemic [3]. In South Korea, the BCPR rate prior to the pandemic was approximately 24.7% and it remained relatively stagnant at 26.0-29.0% during the pandemic [4], with no clear evidence of full recovery post-pandemic. Survival outcomes similarly declined, with international BCPR-related survival rates dropping from 22.1-38.6% pre-pandemic [5] to nearly half during the pandemic [6-8]. Domestically, the survival rate fell from 15.0% to approximately 11-12% and continues to remain low [4].

While previous studies have explored cardiopulmonary resuscitation (CPR) willingness during or long after the pandemic, limited research has examined public attitudes and behaviors in the immediate post-pandemic period - a time when health behaviors are in flux and new societal norms are forming. Existing literature indicates that the willingness to perform CPR varies significantly across different occupational groups. For instance, studies comparing the general public, healthcare professionals (medical doctors and nurses), and care workers have found that non-medical personnel (the general public and care workers) exhibit lower willingness and confidence in performing CPR, and are more likely to hesitate due to concerns about technique [9]. Similarly, non-clinical hospital employees, such as administrative and support staff, have demonstrated limited CPR skill acquisition [10]. In addition, non-medical staff working in medical school settings have been found to possess insufficient CPR knowledge, despite exhibiting positive attitudes toward resuscitation [11].

Numerous factors influence the performance of BCPR, with lack of knowledge consistently identified as a key barrier [12]. In response to the COVID-19 pandemic, the American Heart Association (AHA) issued interim guidelines in April 2020, recommending compression-only CPR for lay rescuers to mitigate infection risks [13]. Thus, it is important to assess awareness of these modified guidelines among potential responders. Despite the official conclusion of the pandemic, sporadic outbreaks of COVID-19 continue to occur, and residual concern regarding infection risk may still influence individual willingness to perform BCPR.

Understanding behavioral changes in the immediate post-pandemic context is essential to evaluate how emergency response behaviors recover or remain suppressed following large-scale public health emergencies. Such insights are critical for strengthening the sustainability and resilience of the community-based emergency medical response system. By identifying factors associated with the willingness to perform BCPR, this study aims to provide foundational evidence to support the development of targeted intervention strategies to enhance BCPR provision and improve OHCA survival outcomes, particularly in the face of future public health threats.

Objectives

This study aims to investigate the determinants of willingness to perform BCPR among hospital non-clinical personnel during the immediate post-COVID-19 period. The specific objectives are to examine the general demographic characteristics of hospital non-clinical personnel, to assess CPR-related characteristics of hospital non-clinical personnel, to evaluate the level of willingness to perform BCPR, to measure knowledge pertaining to compression-only CPR, to assess self-reported confidence in performing CPR, to explore attitudes toward CPR provision, and to identify and analyze factors associated with the willingness to perform BCPR.

## Materials and methods

Study design

This descriptive study aims to identify the factors influencing the willingness of hospital non-clinical personnel to perform BCPR. The descriptive research design was selected to facilitate a comprehensive understanding of the characteristics, knowledge, and attitudes of hospital non-clinical personnel in relation to BCPR.

Participants

The study sample comprised hospital non-clinical personnel employed at Konkuk University Medical Center, including administrative staff, information technology (IT) professionals, medical equipment and facility technicians, patient transport personnel, hospital security officers, support staff, and environmental services personnel. Care workers and nursing assistants were excluded from the study due to their indirect involvement in the clinical care and management of patients.

Sample size estimation was conducted using G*power version 3.1.9.4 (Heinrich-Heine-Universität Düsseldorf, Düsseldorf, Germany), with parameters set at an effect size of 0.15, a significance level (α) of 0.05, statistical power (1-β) of 0.95, and 19 predictor variables for multiple linear regression analysis. Based on these criteria, a minimum sample size of 217 participants was calculated. To account for a potential attrition rate of 5-10%, the target recruitment range was selected between 228 and 239 participants. Inclusion criteria included adults aged 19 years or older who voluntarily agreed to participate and were able to read and comprehend Korean. Predictor variables related to willingness to perform CPR included general demographic and CPR-related characteristics, knowledge of compression-only CPR, CPR performance confidence, and CPR-related attitudes. Of the 230 participants who completed the survey, 15 were excluded for providing insincere responses, resulting in a final sample size of 215.

Research variables

A structured questionnaire was utilized in this study, with all measurement instruments employed under the permission of their original authors. The items assessing participants’ general and CPR-related characteristics were developed based on an extensive review of the relevant literature [3,14-16].

General demographic characteristics included age, sex, marital status, education level, monthly income, occupational role, presence or absence of comorbid conditions, types of comorbidities, family structure, and self-perceived physical health status. CPR-related characteristics encompassed prior CPR training experience, exposure to real-life CPR situations, previous CPR performance, preferred method of CPR delivery, awareness of the locations of automated external defibrillators (AEDs) in public spaces, and exposure to CPR and AED use through various media sources (Appendices).

Knowledge of Performing CPR

Knowledge of CPR performance was measured using an eight-item questionnaire originally developed by Karuthan et al. [3] to assess knowledge of compression-only CPR. Each item was scored dichotomously, with one point assigned for a correct response and zero points for incorrect or “do not know” responses. Total score ranged from 0 to 8, with higher scores indicating greater knowledge of CPR performance. In the present study, the internal consistency reliability of the instrument, as assessed by the Kuder-Richardson formula 20 (KR-20), was 0.51. Details of the questions and response options assessing knowledge of performing CPR are provided in the questionnaire included in the appendix.

Confidence in Performing CPR

Confidence in performing CPR was evaluated using the three items adapted from the instrument developed by Sasaki et al. [14], which assess confidence in performing chest compressions, administering mouth-to-mouth resuscitation, and operating an AED. Each item was rated on a five-point Likert scale (1 = not confident at all to 5 = very confident), with higher total scores reflecting greater self-reported confidence in performing CPR-related tasks.

Attitude in Performing CPR

CPR-related attitudes were assessed using a dichotomous (yes/no) item adapted from Chong et al. [17], which asked participants whether the COVID-19 pandemic had a negative impact on their willingness to perform CPR.

Willingness to Perform CPR

Willingness to perform CPR was assessed using a five-item instrument developed by Karuthan et al. [3], which evaluates participants’ willingness to administer CPR to individuals in various scenarios, including strangers, trauma patients, children, older adults, family members, and relatives. Each item required a dichotomous (yes/no) response. Participants who responded “no” to any scenario were asked to indicate their reasons, which could include lack of knowledge or skill, fear of causing harm, concerns about disease transmission, fear of legal consequences, and cultural or religious beliefs [3]. The internal consistency reliability of the instrument, as measured by the Kuder-Richardson formula 20 (KR-20), was 0.81 in this study. Additionally, willingness to perform CPR was assessed using a five-point Likert scale in response to the question, “If a patient with suspected cardiac arrest were in front of you, would you perform CPR?”. A score of 1 indicated “Would never perform CPR,” while a score of 5 indicated “Would definitely perform CPR.”

Data collection

Data collection was conducted from July 27 to August 13, 2023. The official end of the COVID-19 pandemic in South Korea was declared on May 11, 2023. Therefore, the period within three months following this date was designated as the immediate post-COVID-19 period.

Participants were recruited through word-of-mouth and approached at the time of their attendance at in-hospital CPR training sessions. Prior to participation, the purpose and procedures of the study were explained verbally, and written informed consent was obtained. A structured, paper-based questionnaire was administered to hospital non-clinical personnel attending the training. Completion of the survey required approximately five to 10 minutes. As a token of appreciation, participants received a small incentive in the form of a coffee voucher.

Data analysis

Data analysis was performed using R version 4.2.0 (The Comprehensive R Archive Network [18], Auckland, New Zealand).

Descriptive statistics, including frequencies, percentages, means, and standard deviations, were used to summarize participants’ general demographic and CPR-related characteristics, knowledge of compression-only CPR, self-reported confidence in CPR performance, attitudes toward CPR, and willingness to perform CPR. Willingness to perform CPR, measured on a five-point Likert scale, was dichotomized based on the median value of 3. Participants scoring 0-2 were classified as having low willingness, while those scoring 3-5 were classified as having high willingness. The characteristics of these two groups were summarized using means and standard deviations for continuous variables, and frequencies and percentages for categorical variables. Group comparisons were conducted using independent samples t-tests for continuous variables and chi-square tests for categorical variables. To identify the factors associated with willingness to perform CPR, a multiple linear regression analysis was conducted, using the mean score of the five-point Likert scale scores for willingness to perform CPR as the dependent variable. Prior to performing the regression, diagnostic tests were conducted to evaluate key statistical assumptions. Multicollinearity among independent variables was assessed using tolerance values and variance inflation factors (VIFs). Tolerance values ranged from 0.11 to 0.15 (all > 0.10), and VIF values ranged from 1.02 to 1.29 (all < 10), indicating that multicollinearity was not present. The independence of residuals was examined using the Durbin-Watson statistic, which yielded a value of 2.06 (p = 0.651), suggesting no evidence of autocorrelation. All statistical tests were two-tailed, with a significance level set at p-value < 0.05.

Ethical considerations

This study was approved by the Institutional Review Board of Konkuk University Hospital (KUMC 2023-07-030). Prior to participation, all individuals were provided with a detailed explanation of the study’s purpose, procedures, and ethical safeguards. Informed consent was obtained from those who voluntarily agreed to participate. Participants were assured of the confidentiality and anonymity of their responses and were informed that their participation was entirely voluntary, with the right to decline or withdraw from the study at any point without any consequences. The details were clearly outlined in the informed consent documentation. All collected data were anonymized and securely stored on a password-protected computer. In accordance with institutional data management protocols, the data will be retained for a period of three years and subsequently destroyed to ensure confidentiality.

## Results

General characteristics

The mean age of participants was 37.31 ± 10.84 years, with approximately 60% of them in their 20s or 30s. In the low-willingness group, the largest proportion were in their 30s, whereas in the high-willingness group, the largest proportion were in their 20s. Notably, no participants aged ≥ 60 years were observed in the high-willingness group (χ² = 20.76, p < 0.001). Regarding sex distribution, 45.6% were male and 54.4% were female, with no significant difference in willingness to perform CPR by sex (χ² = 0.01, p = 0.924). Marital status was evenly distributed (49.3% single, 48.8% married), and no significant association was found with willingness to perform CPR (χ² = 1.63, p = 0.443). Most participants (84.7%) had attained a college-level education or higher, with no significant difference between willingness groups (χ² = 0.62, p = 0.429). Over half (51.6%) reported a monthly income exceeding five million Korean won, and 53.5% were employed full-time. The most common occupational categories were office staff (38.6%) and technical staff (34.9%), followed by patients transport personnel, hospital security officers, support staff, and environmental services personnel. Monthly income (χ² = 3.71, p = 0.295), employment type (χ² = 0.60, p = 0.444), and occupational category (χ² = 4.90, p = 0.086) did not differ significantly between groups in the willingness to perform CPR. Comorbid conditions were reported by 22.3% of participants, with hypertension (33.3%), diabetes mellitus (18.8%), cancer (12.5%), and hyperlipidemia (12.5%) being the most prevalent. No significant difference in comorbidity prevalence was observed between groups (χ² = 2.22, p = 0.136). A total of 84.7% lived with family members; among these, 14.0% lived with preschool-aged children and 17.2% with older adults. Of those living with older adults, 12.6% reported that the older adult had comorbidities, most commonly hypertension (70.4%) and diabetes mellitus (51.9%). None of these living arrangement variables differed significantly between willingness groups (χ² = 0.54, p = 0.462; χ² = 0.06, p = 0.807; χ² = 0.01, p = 0.906; χ² = 0.91, p = 0.339). Self-perceived physical health status averaged 6.72 ± 1.66 on a 10-point scale. Participants in the high-willingness group reported significantly higher scores (6.96 ± 1.63) compared with the low-willingness group (6.40 ± 1.65; t = -2.46, p = 0.015) (Table 1).

**Table 1:** General demographic characteristics SD = standard deviation

Variables	Categories	n (%) or mean ± SD	Low-willingness group (n = 94)	High-willingness group (n = 121)	χ² or t (p)
Age		37.31 ± 10.84	37.38 ± 11.56	37.25 ± 10.29	0.09 (0.928)
	20s	63 (29.3)	23 (24.5)	40 (33.1)	20.76
	30s	63 (29.3)	36 (38.3)	27 (22.3)	
	40s	57 (26.5)	21 (22.3)	36 (29.7)	
	50s	24 (11.2)	6 (6.4)	18 (14.9)	
	≥ 60s	8 (3.7)	8 (8.5)	0	
Sex	Male	98 (45.6)	42 (44.7)	56 (46.3)	0.01 (0.924)
	Female	117 (54.4)	52 (55.3)	65 (53.7)	
Marital state	Single	106 (49.3)	46 (48.9)	60 (49.6)	1.63 (0.443)
	Married	105 (48.8)	45 (47.9)	60 (49.6)	
	Other	4 (1.9)	3 (3.2)	1 (0.8)	
Education level	≤ High school	33 (15.3)	17 (18.1)	16 (13.2)	0.62 (0.429)
	≥ College	182 (84.7)	77 (81.9)	105 (86.8)	
Average monthly income (million South Korean won)	1.99	3 (1.4)	0	3 (2.5)	3.71 (0.295)
	2.00-2.99	45 (21.0)	22 (23.4)	23 (19.0)	
	3.00-5.00	54 (25.1)	26 (27.6)	28 (23.1)	
	5.00	111 (51.6)	45 (47.9)	66 (54.6)	
	No response	2 (0.9)	1 (1.1)	1 (0.8)	
Type of employment	Full-time employee	115 (53.5)	47 (50.0)	68 (56.2)	0.60 (0.444)
	Other	100 (46.5)	47 (50.0)	53 (43.8)	
Type of job	Office staff	83 (38.6)	32 (34.0)	51 (42.1)	4.90 (0.086)
	Technical staff	75 (34.9)	30 (31.9)	45 (37.2)	
	Other	57 (26.5)	32 (34.0)	25 (20.7)	
Comorbidity	Yes	48 (22.3)	26 (27.7)	22 (18.2)	2.22 (0.136)
	No	167 (77.7)	68 (72.3)	99 (81.8)	
Type of comorbidity (duplicate)	Hypertension	16 (33.3)	10 (38.5)	6 (27.3)	-
	Diabetes mellitus	9 (18.8)	3 (11.5)	6 (27.3)	-
	Cancer	6 (12.5)	3 (11.5)	3 (16.6)	-
	Hyperlipidemia	6 (12.5)	5 (19.2)	1 (4.5)	-
	Cardiovascular	3 (6.3)	2 (7.7)	1 (4.5)	-
	Kidney disease	2 (4.2)	0	2 (27.3)	-
	Mental illness	2 (4.2)	1 (3.8)	1 (4.5)	-
	Other	13 (27.1)	8 (30.8)	5 (22.7)	-
Cohabiting family	Yes	182 (84.7)	82 (87.2)	100 (82.6)	0.54 (0.462)
	No	33 (15.3)	12 (12.8)	21 (17.6)	
Children in cohabiting families	Yes	30 (14.0)	12 (12.8)	18 (14.9)	0.06 (0.807)
	No	185 (86.0)	82 (87.2)	103 (85.1)	
Elderly in cohabiting families	Yes	37 (17.2)	17 (18.1)	20 (16.5)	0.01 (0.906)
	No	178 (82.8)	77 (81.9)	101 (83.5)	
Comorbidity among the elderly in cohabiting families	Yes	27 (12.6)	9 (9.6)	18 (14.9)	0.91 (0.339)
	No	188 (87.4)	85 (90.4)	103 (85.1)	
Type of comorbidity (duplicate)	Hypertension	19 (70.4)	7 (77.8)	12 (66.7)	-
	Diabetes mellitus	14 (51.9)	5 (55.6)	9 (50.0)	-
	Cardiovascular	6 (22.2)	3 (33.3)	3 (16.7)	-
	Cancer	4 (14.8)	0	4 (22.2)	-
	Hyperlipidemia	2 (7.4)	1 (11.1)	1 (5.6)	-
	Other	3 (11.1)	0	3 (16.7)	-
Self-perceived physical health status	-	6.72 ± 1.66	6.40 ± 1.65	6.96 ± 1.63	-2.46 (0.015)

CPR-related characteristics

The largest proportion of participants (33.5%) reported receiving CPR training within the past one to three years. The proportion of participants with no prior CPR training was approximately four times higher in the low-willingness group compared with the high-willingness group (χ² = 15.50, p = 0.001). Overall, 34.0% had previously encountered a situation requiring CPR, while only 3.3% had personally performed CPR. No significant difference between willingness groups was observed for prior CPR situation exposure (χ² = 0.17, p = 0.681) or direct CPR performance experience (χ² = 0.19, p = 0.664). Chest compression-only CPR was the most preferred resuscitation method (71.6%). A higher proportion of participants in the high-willingness group reported preferring both chest compression-only CPR and CPR combining chest compressions with rescue breaths (χ² = 14.25, p = 0.001). Regarding AEDs, 34.4% indicated that they were aware of AED locations in public places. This proportion was significantly higher in the high-willingness group (χ² = 13.83, p < 0.001). In addition, 82.8% reported having been exposed to information about CPR techniques and AED use through various media sources; however, no significant difference was observed between willingness groups in this regard (χ² = 2.48, p = 0.115) (Table 2).

**Table 2:** CPR-related characteristics AED = automated external defibrillator; CPR = cardiopulmonary resuscitation

Variables	Categories	n (%)	Low-willingness group (n = 94)	High-willingness group (n = 121)	χ² or t (p)
Previous CPR training	Less than one year	67 (31.2)	24 (25.5)	43 (35.6)	15.50 (0.001)
	One to three years	72 (33.5)	25 (26.6)	47 (38.8)	
	More than three years	46 (21.4)	23 (24.5)	23 (19.0)	
	No experience	30 (13.9)	22 (23.4)	8 (6.6)	
Experience with CPR situation	Yes	73 (34.0)	30 (31.9)	43 (35.5)	0.17 (0.681)
	No	142 (66.0)	64 (68.1)	78 (64.5)	
CPR performance experience	Yes	7 (3.3)	2 (2.1)	5 (4.1)	0.19 (0.664)
	No	208 (96.7)	92 (97.9)	116 (95.9)	
Preferred method of performing CPR	Chest compression and mouth-to-mouth	42 (19.5)	15 (16.0)	27 (22.3)	14.25 (0.001)
	Hands-only CPR	154 (71.6)	63 (67.0)	91 (75.2)	
	Not selected	19 (8.8)	16 (17.0)	3(2.5)	
Awareness of the location of AED in public place	I know	74 (34.4)	19 (31.9)	55(35.5)	13.83
	I don’t know	141 (65.6)	75 (68.1)	66 (64.5)	
Receiving information about CPR or AED through the media	Yes	178 (82.8)	73 (77.7)	105 (86.8)	2.48 (0.115)
	No	37 (17.2)	21 (22.3)	16 (13.2)	

Knowledge of compression-only CPR and confidence and attitude toward CPR

The mean knowledge score for compression-only CPR was 5.76 ± 1.46 out of a maximum of eight points. The lowest correct response rate (19.1%) was observed for the item assessing the correct sequence of CPR; most participants selected the traditional sequence, including artificial ventilation, rather than the recommended compression-only approach. The second lowest correct response rate (62.8%) was for the correct chest compression location, with many respondents incorrectly identifying the upper chest instead of the center of the chest. The correct response rate for the compression rate was 64.7%. Participants in the high-willingness group had significantly higher knowledge scores (5.97 ± 1.43) compared with those in the low-willingness group (5.49 ± 1.45; t = -2.41, p = 0.017). Item-level analysis revealed that the low-willingness group had a higher correct response rate for chest compression location, whereas the high-willingness group performed better on items assessing correct chest compression rate and depth.

Confidence in performing CPR was generally low, with a mean score of 5.81 ± 3.59 out of a possible 15 points. The high-willingness group demonstrated substantially greater confidence (7.51 ± 3.26) compared with the low-willingness group (4.10 ± 3.38; t = -7.49, p < 0.001).

Regarding attitude toward CPR, 65.6% of participants reported that the COVID-19 pandemic had not negatively affected their willingness to perform CPR. No significant difference in attitudes was observed between willingness groups (χ² = 0.39, p = 0.534) (Table 3).

**Table 3:** Descriptive statistics for measured variable CPR = cardiopulmonary resuscitation; SD = standard deviation

Variable	Categories	n (%) or mean ± SD	Low-willingness group (n = 94)	High-willingness group (n = 121)	χ² or t (p)
CPR knowledge	0-8 (min-max)	5.76 ± 1.46	5.49 ± 1.45	5.97 ± 1.43	-2.41 (0.017)
Items					
1. Can hands-only CPR be performed outside a hospital setting?	Correct	193 (89.8)	83 (88.3)	110 (90.9)	-
	Non-correct	22 (10.2)	11 (11.7)	11 (9.1)	-
2. Can bystander hands-only CPR be performed without mouth-to-mouth resuscitation?	Correct	172 (80.0)	69 (73.4)	103 (85.1)	-
	Non-correct	43 (20.0)	25 (26.6)	18 (14.9)	-
3. Can a person perform hands-only CPR without certification?	Correct	194 (90.2)	79 (84.0)	115 (95.0)	-
	Non-correct	21 (9.8)	15 (16.0)	6 (5.0)	-
4. What is the correct sequence to perform CPR?	Correct	41 (19.1)	13 (13.8)	28 (23.1)	-
	Non-correct	174 (80.9)	81 (86.2)	93 (76.9)	-
5. When should one perform hands-only CPR?	Correct	208 (96.7)	91 (96.8)	117 (96.7)	-
	Non-correct	7 (3.3)	3 (3.2)	4 (3.3)	-
6. Where is the right location to perform chest compression?	Correct	135 (62.8)	66 (70.2)	69 (57.0)	-
	Non-correct	80 (37.2)	28 (29.8)	52 (43.0)	-
7. How fast should one perform chest compression?	Correct	139 (64.7)	53 (56.4)	86 (71.1)	-
	Non-correct	76 (35.3)	41 (43.6)	35 (28.9)	-
8. What is the correct depth of compression that should be performed during chest compression?	Correct	156 (72.6)	62 (66.0)	94 (77.7)	-
	Non-correct	59 (27.4)	32 (34.0)	27 (22.3)	-
CPR confidence	3-15 (min-max)	5.81 ± 3.59	4.10 ± 3.38	7.51 ± 3.26	-7.49
Attitude toward CPR	Negative	74 (34.4)	35(37.2)	39 (32.2)	0.39 (.534)
	Not changed	141 (65.6)	59 (62.8)	8267.8	-

Willingness to perform CPR

The mean score for willingness to perform CPR across five different recipient scenarios was 2.84 ± 1.67 out of a maximum of five points. The proportion of participants indicating willingness to perform CPR varied by recipient type: 92.6% for family members, 61.9% for strangers, 53.0% for children, 51.2% for older adults, and 25.6% for trauma patients. Among the barriers to performing CPR, the most frequently reported concern across scenarios involving trauma patients, children, and older adults was the fear of causing harm, cited by 50% to 75% of respondents. In contrast, when considering CPR performance on family members, the most commonly reported barrier was a perceived lack of knowledge and skills, reported by 81.3% of participants (Table 4).

**Table 4:** CPR willingness CPR = cardiopulmonary resuscitation; SD = standard deviation

Variable	Categories	n (%) or mean ± SD
Total		2.84 ± 1.67
Stranger (duplicate)	Yes	133 (61.9)
	No	82 (38.1)
	No, because of poor knowledge/technique	40 (48.8)
	No, because I am afraid of hurting the victim	40 (48.8)
	No, because I am afraid of disease transmission	11 (13.4)
	No, because I am afraid of legal issues	36 (43.9)
	No, because of social/religious reasons	1 (1.2)
	Others	0
Trauma victim (duplicate)	Yes	55 (25.6)
	No	160 (74.4)
	No, because of poor knowledge/technique	56 (35.0)
	No, because I am afraid of hurting the victim	122 (76.3)
	No, because I am afraid of disease transmission	8 (5.0)
	No, because I am afraid of legal issues	39 (24.4)
	No, because of social/religious reasons	1 (0.6)
	Others	7 (4.4)
Child (duplicate)	Yes	114 (53.0)
	No	101 (47.0)
	No, because of poor knowledge/technique	53 (52.5)
	No, because I am afraid of hurting the victim	68 (67.3)
	No, because I am afraid of disease transmission	3 (3.0)
	No, because I am afraid of legal issues	37 (36.6)
	No, because of social/religious reasons	0
	Others	1 (1.0)
Elderly person (duplicate)	Yes	110 (51.2)
	No	105 (48.8)
	No, because of poor knowledge/technique	46 (43.8)
	No, because I am afraid of hurting the victim	79 (75.2)
	No, because I am afraid of disease transmission	7 (6.7)
	No, because I am afraid of legal issues	36 (34.3)
	No, because of social/religious reasons	0
	Others	0
Family (duplicate)	Yes	199 (92.6)
	No	16 (7.4)
	No, because of poor knowledge/technique	13 (81.3)
	No, because I am afraid of hurting the victim	7 (43.8)
	No, because I am afraid of disease transmission	0
	No, because I am afraid of legal issues	1 (6.3)
	No, because of social/religious reasons	0
	Others	1 (6.3)

Factors associated with willingness to perform CPR

This regression analysis revealed that higher confidence in performing CPR, female sex, and prior CPR training were significant positive predictors of willingness to perform CPR. The overall regression model was statistically significant (F = 16.49, p < 0.001), explaining approximately 31% of the variance in willingness to perform CPR (R² = 0.31) (Table 5).

**Table 5:** Factors related to willingness to perform CPR AED = automated external defibrillator; CPR = cardiopulmonary resuscitation; SE = standard error

Variables	B	SE	β	t	p
CPR confidence	0.22	0.03	0.47	7.25	<0.001
Female	0.44	0.21	0.13	2.14	0.034
Education level (≥ college)	0.47	0.27	0.10	1.73	0.084
Average monthly income (5.00 million won)	0.24	0.19	0.07	1.24	0.217
Previous CPR training	0.75	0.31	0.16	2.46	0.014
Awareness of the location of AED	0.38	0.21	0.11	1.83	0.068
F = 16.49 (p < 0.001)					

## Discussion

This study identified key factors influencing hospital non-clinical personnel’s willingness to perform BCPR in the immediate post-COVID-19 period, including CPR-related knowledge, confidence, attitude, demographic, and experiential variables.

The analysis of willingness to perform CPR across various scenarios revealed that 61.9% of participants were willing to assist strangers, 25.6% trauma patients, 53.0% children, 51.2% older adults, and 92.6% family members. Compared to pre-pandemic findings, which reported lower willingness rates across most group-55% for strangers, 37.4% for trauma patients, 45% for children, 49.1% for older adults, and 67.7% for family members [3] - this study suggests a recovery or even an increase in willingness post-pandemic, with the exception of trauma patients. These findings are consistent with international trends. For example, a Thai study indicated a decline in willingness to perform CPR during the COVID-19 pandemic [19], which has since rebounded. While direct comparison across studies is limited by differences in methodology, populations, and local pandemic impact, the overall trend implies a restoration of public confidence in performing CPR. This may be attributed to increased exposure to OHCA cases during the COVID-19 pandemic, coupled with heightened awareness of and appreciation for the value of life following the widespread experience of mortality during the crisis.

Nonetheless, fear of inflicting harm, harm-particularly to vulnerable groups like trauma patients, children, and older adults, emerged as a primary deterrent, consistent with previous research indicating apprehension over potential physical injury and associated legal liabilities [12,20]. In South Korea, legal protection for bystanders performing emergency care is provided under Article 5-2 of the Emergency Medical Services Act, commonly referred to as the Good Samaritan Law [21]. However, despite this legal safeguard, concerns about potential legal liability persist among the public. As evidenced by prior studies [22], even CPR training does not significantly reduce fears of legal repercussions. Thus, public education campaigns should incorporate information about legal safeguards to alleviate these concerns and promote responsible action during emergencies.

Greater self-reported confidence in performing CPR, prior CPR training, and female sex were identified as positive predictors of willingness to perform BCPR. Notably, participants in the high-willingness group exhibited significantly higher confidence levels than those in the low-willingness group. Furthermore, the proportion of individuals without prior CPR training was approximately fourfold higher in the low-willingness group compared with the high-willingness group. These findings align with existing literature demonstrating that confidence is strongly associated with both CPR competence and willingness to act [16,17]. While debates remain regarding optimal training intervals, some studies suggest that willingness and confidence peak when training occurs within the previous year [20] and diminish beyond three to 12 months post-training [23]. Accordingly, recurring CPR and AED training, particularly for healthcare support personnel, is essential [24,25]. Incorporating simulation-based or hybrid (online and in-person) training models, as developed during the COVID-19 pandemic, may improve engagement and retention.

Interestingly, female participants in this study reported greater willingness to perform BCPR-contrary to previous studies where male respondents typically demonstrated higher willingness, possibly due to perceived physical advantages [26-28]. However, higher empathy and communication skills, often associated with female respondents [26,27], may enhance motivation and confidence, particularly when supported by tailored, scenario-based training. This suggests that gender-sensitive training approaches may be beneficial in optimizing BCPR outcomes.

Notably, CPR knowledge and COVID-19-related attitudes were not significantly associated with willingness to perform CPR. The relatively low knowledge scores observed in this study may be attributed to widespread confusion between traditional CPR sequences, which include ventilation, and chest compression-only protocols. This knowledge gap suggests a need for targeted educational efforts emphasizing compression-only CPR among lay responders. Previous studies indicating a link between knowledge and willingness [12] may have assessed broader or more clearly defined CPR knowledge domains.

Similarly, fear of COVID-19 infection did not significantly impact CPR willingness in this study. This result is consistent with some earlier findings [29,30], though it contrasts with others that cited infection risk as a major barrier [17,31]. Notably, only 34.4% of respondents in this study reported a negative change in attitude toward BCPR post-pandemic, compared to 61.0% of healthcare professionals during the pandemic [17]. This likely reflects reduced perceived risk following the official end of the COVID-19 crisis and suggests a resilient attitude among hospital non-clinical personnel. Their continued willingness to act in the face of emerging infectious threats highlights the importance of supporting these individuals through legal protections and systemic preparedness.

Despite the strengths of this study, several limitations warrant consideration. First, the study sample was restricted to hospital non-clinical personnel at a single institution, limiting generalizability. Future studies should expand to include diverse occupational and regional groups, including students and community volunteers. Second, the cross-sectional design precludes inference about temporal changes in willingness. Longitudinal research is needed to evaluate trends in CPR-related attitudes and behavior over time. In this study, recruited participants who voluntarily enrolled prior to receiving mandatory CPR training for hospital non-clinical personnel, the possibility of selection bias cannot be entirely excluded. Finally, as this study utilized a self-reported questionnaire, the possibility of social desirability bias cannot be ruled out, and the findings may reflect subjective responses. In particular, the instrument used to assess CPR performance knowledge demonstrated low internal reliability. Therefore, future studies should employ instruments with stronger validity and reliability, and additionally consider incorporating objective measures to directly assess actual CPR performance levels.

## Conclusions

This study examined the willingness of hospital non-clinical personnel to perform BCPR in the immediate post-COVID-19 pandemic period and identified key influencing factors influencing this willingness. Willingness varied depending on the victim, with the lowest for trauma patients and the highest for family members. Fear of causing harm was a major barrier, while self-reported confidence, prior CPR training, and being female were associated with increased willingness.

As the willingness to perform BCPR recovers post-pandemic, it remains vulnerable to future health crises. Strengthening CPR training, addressing psychological barriers, and maintaining public education and institutional support are essential to ensuring sustained BPR readiness and improving survival outcomes from OHCA.

## Appendices

**Table 6:** Questionnaire forms used in the study

Knowledge on hands-only CPR
1. Can hands-only CPR be performed outside a hospital setting?
□ Yes
□ No
□ Not sure
2. Can bystander hands-only CPR be performed without mouth-to-mouth resuscitation?
□ Yes
□ No
□ Not sure
3. Can a person perform hands-only CPR without certification?
□ Yes
□ No
□ Not sure
4. What is the correct sequence to perform CPR?
□ Circulation > airway > breathing
□ Airway > breathing > circulation
□ Not sure
5. When should one perform hands-only CPR?
□ When a person is unconscious
□ When a person is not breathing
□ When a person complains of chest pain
□ When there is no pulse felt
□ When a person looks pale/blue
□ When a person complains of feeling dizzy
□ Not sure
6. Where is the right location to perform chest compression?
□ A (Middle of chest)
□ B (Under the left clavicle)
□ C (Above the middle of chest)
□ D (Under the right clavicle)
□ Not sure
7. How fast should one perform chest compression?
□ 150 compressions per minute
□ 100 compressions per minute
□ 50 compressions per minute
□ As fast as possible
□ Not sure
8. What is the correct depth of compression that should be performed during chest compression?
□ Such that the rib cage moves down 1 to 2 cm
□ Such that the rib cage moves down 5 to 6 cm
□ Such that the rib cage moves down 6 to 10 cm
□ As deep as possible
□ Not sure
Confidence in performing CPR
1. Please check the number to indicate your level of confidence performing chest compression.
① (Not confident at all) ② ③ ④ ⑤ (Very confident)
2. Please check the number to indicate your level of confidence performing mouth-to-mouth rescue breathing.
① (Not confident at all) ② ③ ④ ⑤ (Very confident)
3. Please check the number to indicate your level of confidence using AED.
① (Not confident at all) ② ③ ④ ⑤ (Very confident)
Attitude in performing CPR
1. The outbreak of an emerging infectious disease such as COVID-19 has a NEGATIVE impact on my attitude toward bystander CPR
□ No
□ Yes
Willingness to perform hands-only CPR
If a patient with suspected cardiac arrest were in front of you, would you perform CPR?
① (Would never perform CPR) ② ③ ④ ⑤ (Would definitely perform CPR)
1. In an emergency situation, would you perform hands-only CPR on a stranger?
□ Yes
□ No, because of poor knowledge/technique
□ No, because I am afraid of hurting the victim
□ No, because I am afraid of disease transmission
□ No, because I am afraid of legal issues
□ No, because of social/religious reasons
□ Others
2. In an emergency situation, would you perform hands-only CPR on a victim of trauma?
□ Yes
□ No, because of poor knowledge/technique
□ No, because I am afraid of hurting the victim
□ No, because I am afraid of disease transmission
□ No, because I am afraid of legal issues
□ No, because of social/religious reasons
□ Others
3. In an emergency situation, would you perform hands-only CPR on a child?
□ Yes
□ No, because of poor knowledge/technique
□ No, because I am afraid of hurting the victim
□ No, because I am afraid of disease transmission
□ No, because I am afraid of legal issues
□ No, because of social/religious reasons
□ Others
4. In an emergency situation, would you perform hands-only CPR on an elderly person?
□ Yes
□ No, because of poor knowledge/technique
□ No, because I am afraid of hurting the victim
□ No, because I am afraid of disease transmission
□ No, because I am afraid of legal issues
□ No, because of social/religious reasons
□ Others
5. In an emergency situation, would you perform hands-only CPR on a relative/family member?
□ Yes
□ No, because of poor knowledge/technique
□ No, because I am afraid of hurting the victim
□ No, because I am afraid of disease transmission
□ No, because I am afraid of legal issues
□ No, because of social/religious reasons
□ Others
General characteristics and CPR-related questions
1. Please enter your age.
( ) years
2. Please select your gender.
① Male
② Female
3. Please select your marital status.
① Single
② Married
③ Divorce
④ Bereave
⑤ Others
4. Please select your education level.
① High school graduate or less
② College graduate
③ Graduate school or higher
5. Please select your average monthly income.
① Less than 1.99 million Korean won
② 2.00-2.99 million Korean won
③ 3.00-5.00 million Korean won
④ More than 5.00 million Korean won
6. Please select your type of employment.
① Full-time employee
② Contract worker
③ Others
7. Please select your type of job.
① Office work
② Technical work
③ Others
8. Please check all of the current comorbidities that you have been diagnosed with or are taking medication for (multiple checks are allowed)
① None
② HTN
③ DM
④ Cerebrovascular (stroke, hemorrhage)
⑤ Cardiovascular (myocardial infarction, angina, arrhythmia)
⑥ Cancer
⑦ Kidney disease
⑧ Mental illness (panic disorder, depressive disorder)
⑨ Others ( )
9. How many people live with you?
① None
② One
③ Two
④ Three
⑤ Four
⑥ Five
⑦ More than six
10. How many of your cohabitants are preschool children (elementary school age or younger)?
① None
② One
③ Two
④ Three
⑤ More than four
11. How many of the people living with you are over 65 years old?
① None
② One
③ Two
④ Three
⑤ More than four
12. If you have a person living with you who is over 65 years old, please check all the comorbidities that the person over 65 years old currently has (multiple checks are allowed).
① None
② HTN
③ DM
④ Cerebrovascular (stroke, hemorrhage)
⑤ Cardiovascular (myocardial infarction, angina, arrhythmia)
⑥ Cancer
⑦ Kidney disease
⑧ Mental illness (panic disorder, depressive disorder)
⑨ Others( )
13. Please select the number that best represents your self-perceived physical health status.
① (Very bad) ② ③ ④ ⑤ ⑥ ⑦ ⑧ ⑨ ⑩ (Very good)
14. How long has it been since you last received CPR training?
① Less than one year
② One to three years
③ More than three years
④ No experience
15. Have you ever seen someone in cardiac arrest or have you ever seen someone being given CPR?
① Yes
② No
16. Have you ever personally performed CPR outside of a hospital?
① Yes
② No
17. If you were in a situation where CPR was required, which method would you choose?
① Chest compression and mouth-to-mouth
② Hands-only CPR
③ Not selected
18. Do you know where automated external defibrillators (AEDs) are located in public places outside hospitals?
① Yes
② No
19. Have you ever come across information about CPR or an automated external defibrillator (AED) through external media (television, internet, cell phone, etc.)?
① Yes
② No
